# Evaluating the Sealing Potential of Different Materials for Perforation Repair: An In Vitro Study

**DOI:** 10.7759/cureus.117378

**Published:** 2026-10-03

**Authors:** Kanupriya Kanupriya, Amit Choudhary, Shantun Malhotra, Kanwalpreet K Bhullar, Navchandan Kaur, Monica Monica

**Affiliations:** 1 Department of Conservative Dentistry and Endodontics, Sri Guru Ram Das Institute of Dental Sciences and Research, Amritsar, IND; 2 Department of Orthodontics and Dentofacial Orthopaedics, Maharaja Ganga Singh Dental College and Research Centre, Sri Ganganagar, IND; 3 Department of Paediatric and Preventive Dentistry, Genesis Institute of Dental Sciences and Research, Firozpur, IND; 4 Department of Paediatric and Preventive Dentistry, Private Practitioner, Delhi, IND

**Keywords:** bio-c repair, biodentine, guttaflow bioseal, microleakage, strip perforation

## Abstract

Introduction: Root perforations pose a significant challenge in endodontic treatment, necessitating effective sealing materials to prevent microleakage and ensure long-term success. Advances in biocompatible materials have led to the introduction of various repair options, including injectable bioceramics and conventional powder-liquid systems. This study compared the performance of injectable materials (Bio-C Repair, Angelus, Londrina, Paraná, Brazil; and Guttaflow Bioseal, Coltène/Whaledent, Langenau, Germany) with the conventional powder-liquid system (Biodentine, Septodont S.A.S., Saint-Maur-des-Fossés, France) in minimizing microleakage in strip perforation repairs. The null hypothesis stated that there would be no significant difference in microleakage among the tested materials.

Methods: An in vitro study was conducted using 50 extracted human molars. Root canals were prepared using rotary files, and strip perforations were created in the mesiobuccal canal using Gates-Glidden drills. The teeth were divided into five groups of 10 samples each, including three experimental groups (Biodentine, Bio-C Repair, and Guttaflow Bioseal) and two control groups. We assessed microleakage using the dye extraction method and Ultraviolet-Visible spectrophotometry at 550 nm. Statistical analysis was performed using analysis of variance and the Tukey post hoc test, with p < 0.05 considered significant.

Results: Statistically significant differences in microleakage were observed among the groups. Among the experimental groups, Bio-C Repair exhibited the least microleakage, followed by Guttaflow Bioseal and Biodentine.

Conclusion: The findings indicate that injectable root repair materials, such as Bio-C Repair and Guttaflow Bioseal, outperform the conventional powder-and-liquid system of Biodentine in minimizing microleakage. These materials present potential advantages for clinical applications requiring effective root repair.

## Introduction

Root perforation is an undesirable procedural complication of endodontic treatment and is an important cause of treatment failure. It represents an artificial communication between the root canal system and the periodontal tissues, which may result from excessive instrumentation, inappropriate canal preparation, or iatrogenic use of rotary and hand instruments [1-3]. Among the different types of perforations, strip perforations are particularly challenging because they usually occur along the thin lateral aspect of the root where the dentinal wall is already structurally compromised. If left untreated or inadequately sealed, the communication provides a pathway for bacterial contamination and inflammatory destruction of the surrounding periodontal tissues, adversely affecting the prognosis of the tooth [4,5].

The mesial roots of mandibular molars are particularly susceptible to strip perforation because of their characteristic anatomy. These roots frequently contain narrow and curved canals with a relatively thin dentinal wall on the furcal aspect. This region is commonly referred to as the “danger zone” because excessive removal of dentin during canal enlargement can result in weakening or perforation of the root. The danger zone is particularly relevant during coronal and middle-third enlargement when Gates-Glidden (GG) burs are used. Improper selection of bur size, excessive apical pressure, deep penetration, or incorrect angulation of the instrument can result in preferential removal of dentin from the furcal wall and may produce a longitudinal or strip-type perforation [2,3]. Therefore, knowledge of root morphology and controlled instrumentation techniques are essential to prevent this complication.

GG burs are traditionally used for coronal enlargement and straight-line access to root canals. However, their use in curved and anatomically narrow canals requires careful control of depth, direction, and pressure. The excessive use of larger GG burs in the danger zone may cause unnecessary dentin removal and create a strip perforation. In experimental models, standardized strip perforations can be created using GG burs to reproduce this clinically relevant iatrogenic complication and to evaluate the sealing ability of different repair materials [2,3].

Successful management of a perforation depends substantially on the ability of the repair material to establish an effective seal between the root canal system and the surrounding tissues. An ideal perforation repair material should exhibit good sealing ability, dimensional stability, biocompatibility, adequate handling characteristics, resistance to dissolution, and sufficient mechanical stability. Calcium silicate-based materials have gained considerable attention because of their hydraulic setting reaction, bioactivity, alkaline pH, and ability to promote mineralized tissue formation. Biodentine is a tricalcium silicate-based cement developed as a dentin-replacement and repair material. It has been widely used for perforation repair because of its biocompatibility, bioactivity, sealing ability, and relatively rapid setting characteristics [6-8]. However, its powder-and-liquid formulation requires mixing before placement, and its relatively stiff consistency may influence adaptation to small or irregular perforation defects.

Bio-C Repair is a premixed, ready-to-use bioceramic repair material based on calcium silicate chemistry. Its premixed formulation eliminates the need for chairside mixing and may provide consistent handling and placement. The material has also been reported to demonstrate bioactivity and favorable biological responses, with the potential for mineralized tissue formation at the material-dentin interface [9,10]. Its handling characteristics and particle distribution may facilitate adaptation to irregular defects and narrow root spaces.

GuttaFlow Bioseal represents another category of bioactive endodontic material. Unlike Biodentine and Bio-C Repair, which are primarily used as repair materials, GuttaFlow Bioseal is an obturation sealer consisting of a silicone-based matrix containing finely dispersed gutta-percha and bioactive glass/calcium-silicate components. Its flowable nature allows it to spread into irregularities and microscopic spaces within the root canal system. The incorporation of bioactive components may additionally contribute to interfacial mineralization and sealing behavior [11]. However, because GuttaFlow Bioseal is primarily intended as an obturation sealer rather than a bulk perforation repair cement, its performance in experimentally created strip perforations requires specific evaluation.

The sealing ability of perforation repair materials can be assessed using several experimental methods. Dye penetration and dye-extraction techniques are commonly employed in laboratory investigations because they permit quantitative assessment of leakage. In the dye-extraction method, the amount of dye associated with leakage is quantified after extraction from the specimen, with ultraviolet (UV)-visible spectrophotometry providing an objective measurement of dye concentration. This approach allows quantitative comparison of sealing performance across materials under standardized experimental conditions.

Considering the clinical significance of strip perforations, the anatomical vulnerability of the danger zone, and the increasing availability of premixed and bioactive repair materials, comparative evaluation of their sealing ability is warranted. Therefore, the present in vitro study was undertaken to compare the microleakage of Biodentine, Bio-C Repair, and GuttaFlow Bioseal in experimentally created strip perforations using the dye-extraction method and UV-visible spectrophotometry at 550 nm.

Objectives

This study aimed to compare the sealing ability, in terms of microleakage, of Biodentine, Bio-C Repair, and GuttaFlow Bioseal used to repair experimentally created strip perforations in extracted human molars, and to evaluate whether the different material formulations and delivery characteristics of Biodentine, Bio-C Repair, and GuttaFlow Bioseal result in significant differences in microleakage under standardized in vitro conditions.

Null hypothesis

The null hypothesis states that there is no significant difference in microleakage among Biodentine, Bio-C Repair, and GuttaFlow Bioseal when used for the repair of experimentally created strip perforations.

## Materials and methods

This in vitro study was conducted in the Department of Conservative Dentistry and Endodontics at the Sri Guru Ram Das Institute of Dental Sciences and Research, Amritsar. Ethical authorization for the research project was granted by the Institutional Review Board (1226/IDSR/2026). The study aimed to evaluate the sealing ability of different root repair materials-Biodentine™, Bio-C Repair, and GuttaFlow Bioseal (for the repair of strip perforations). The study also specifically analyzed differences between the powder-liquid and injectable forms of these materials in the repair of strip perforations.

Power analysis

The sample size was calculated using the following formula:

$N = \frac{(Z_{\alpha/2})^{2} \times 2s^{2}}{d^{2}}$

where N represents the required sample size, s is the standard deviation (SD) obtained from a previous study, d represents the desired precision or accuracy of the estimate, and $(Z_{\alpha/2})$ represents the standard normal deviate corresponding to the selected level of significance for a two-tailed hypothesis.

The study power was set at 80%. The effect size calculated in the preliminary trial was 9.47. Considering potential errors, the sample size was increased to 10 specimens per group. The study involved five groups, with 10 specimens in each group. Thus, the study used 50 extracted human molars to evaluate microleakage and the sealing ability of root repair materials in strip perforations.

Sample size estimation was performed based on the preliminary trial using a one-way analysis of variance (ANOVA) design with five study groups. The study power was set at 80% (β = 0.20), with a two-sided significance level of 5% (α = 0.05). The effect size obtained from the preliminary trial was 9.47. Based on these parameters, we estimated the minimum sample size and, to account for experimental error and specimen loss, increased it to 10 specimens per group. Thus, five groups with 10 specimens in each group were included, giving a total sample size of 50 extracted human molars.

Sample preparation

Fifty extracted human molars were distributed into three primary experimental groups, each comprising 10 teeth: Group A received Biodentine™ (Septodont, St. Maur-des-Fossés, France), Group B received Bio-C Repair (Angelus, Londrina, Brazil), and Group C received GuttaFlow Bioseal (Coltene, Roeko, Germany). Two control groups were also included: the positive control (Group D), consisting of intact teeth without strip perforations, and the negative control (Group E), consisting of teeth with strip perforations left unsealed (Figure 1). The teeth were disinfected in 5% sodium hypochlorite for 30 minutes to remove soft-tissue debris and were subsequently stored in normal saline until further use.

**Figure 1:**
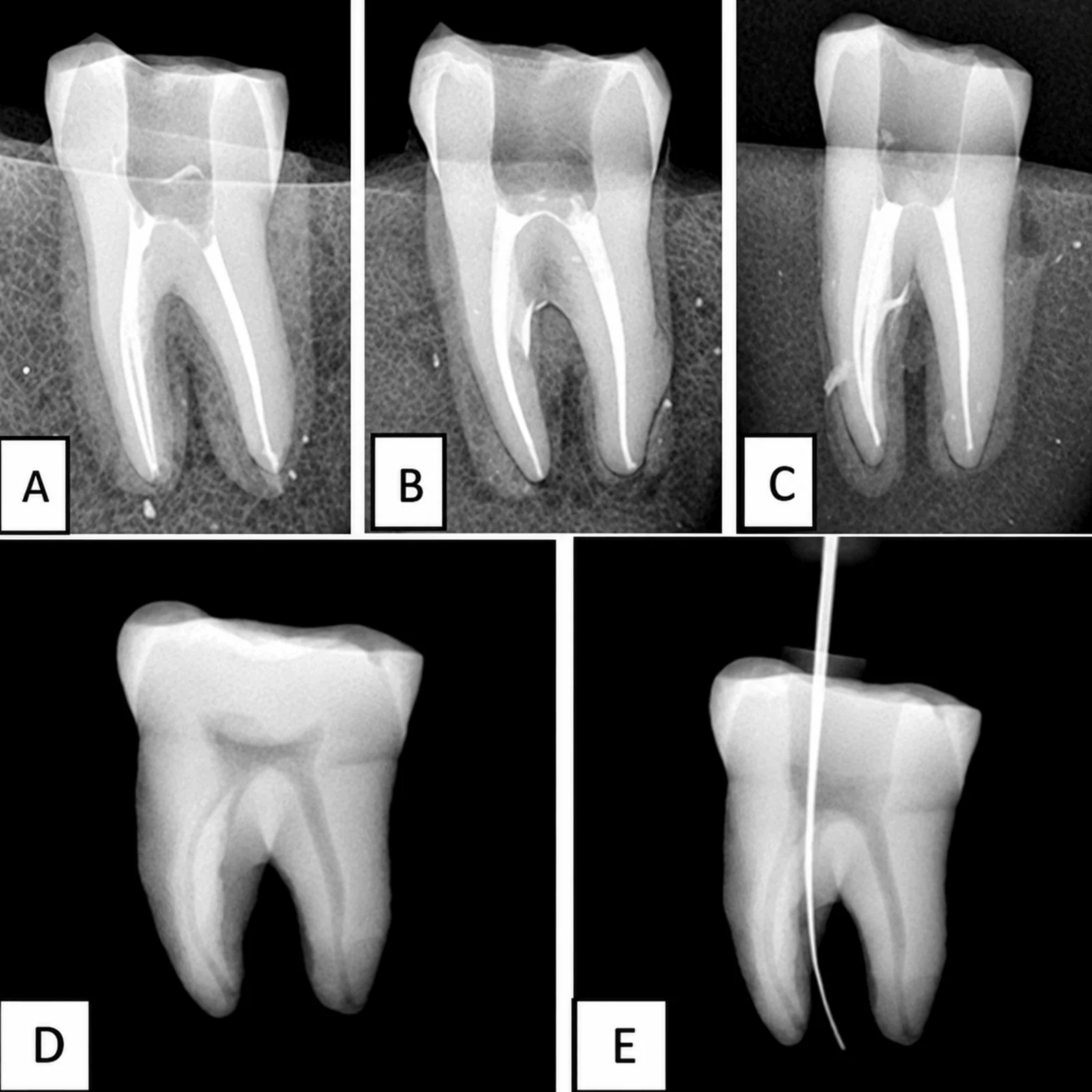
(A) Group A used Biodentine™. (B) Group B used Bio-C Repair. (C) Group C used Guttaflow Bioseal. (D) Positive Control-Group. (D) Consisting intact teeth without strip perforations. (E) Negative Control-Group E, including teeth with strip perforations left unsealed

Conventional access cavities were prepared using an Endo Access Bur (164-014C, Prima Dental, Gloucester, UK) and an Endo-Z bur (Dentsply Tulsa, Tulsa, OK). Biomechanical preparation was performed using the Blueflex Acerfile rotary file system (DenMed, India) up to the working length, with preparation completed to size 25 with a 4% taper. Irrigation was carried out using 5.25% sodium hypochlorite and normal saline. Strip perforations were standardized by marking the mesiobuccal root in the danger zone, approximately 3-4 mm below the furcation.

GG drills (#2 and #3, Mani Inc., Japan) were used in a distoaxial direction to progressively thin the dentin until perforation occurred. Perforation was confirmed by inserting a #20 K-file through the perforation site. The perforation dimensions were standardized as far as possible by using the same drill sizes, instrumentation protocol, depth, and direction for all specimens. The specimens were examined under magnification to ensure comparable perforation morphology before repair. Details regarding the method of standardization and verification have now been incorporated into the Methods section.

To simulate the periodontal ligament, the roots were coated with a 0.2-0.3 mm layer of wax and subsequently embedded in autopolymerizing acrylic resin. A 0.2-mm wax spacer was used circumferentially around the root surface to provide a standardized space for the simulated periodontal ligament. After removal of the wax spacer, polyvinyl siloxane (PVS) was applied to occupy the resulting space, thereby maintaining an approximately uniform 0.2-mm simulated periodontal ligament layer. The uniformity of the PVS layer was assessed visually around the root surface after setting; however, the thickness was not independently quantified at multiple points using digital microscopy or cross-sectional measurement. During placement of Biodentine and Bio-C Repair, the materials were inserted and adapted using controlled, minimal pressure to avoid excessive loading of the external root surface. Care was taken to prevent displacement or deformation of the PVS lining. After the resin had set, the teeth were removed, and the wax was eliminated. The resulting space was coated with PVS before reinserting the teeth into the acrylic blocks. For obturation, moisture was removed using paper points. A single-cone technique was employed using size 25, 4% taper gutta-percha cones (Meta Biomed, Cheongju, South Korea). The mesiobuccal canals were obturated with the respective repair materials, while the distal and mesiolingual canals were obturated with gutta-percha and RC Seal (Prime Dental, India).

In Group A, the Biodentine™ powder was agitated, the liquid container was opened, and five drops of distilled water were added to the capsule. The capsule was mixed for 30 seconds at 4,000-4,200 rpm using an amalgamator. The material was applied using a mineral trioxide aggregate (MTA) carrier and condensed with a gutta-percha instrument. In Group B, Bio-C Repair was directly introduced into the perforation site and condensed using gutta-percha. In Group C, GuttaFlow Bioseal was injected into the perforation site, ensuring adequate sealing. The coronal access cavities were subsequently sealed using composite resin (Te-Econom Plus, Ivoclar Vivadent, UK).

For microleakage evaluation, all tooth surfaces, except the perforation sites, were coated with nail varnish. The samples were immersed in 2% methylene blue dye for 48 hours. The teeth were subsequently washed, dried, and immersed in 65% nitric acid for three days. The samples were then centrifuged at 3,500 rpm for five minutes. A 400 µL aliquot of the supernatant from each sample was analyzed using a UV-visible spectrophotometer at a wavelength of 550 nm.

Statistical analysis

Statistical analysis was performed using IBM Statistical Package for the Social Sciences Statistics for Windows, version 22.0 (IBM Corp., Armonk, NY). Continuous data were expressed as mean ± SD, along with minimum and maximum values and 95% confidence intervals. Because the data followed a normal distribution, we compared mean microleakage values among the five study groups using one-way ANOVA. When ANOVA showed a statistically significant difference, we performed pairwise intergroup comparisons using Tukey's Honestly Significant Difference (HSD) post hoc test. A two-tailed p value of <0.05 was considered statistically significant.

## Results

The mean microleakage values, expressed as optical absorbance in absorbance units (AU), differed significantly among the five study groups, as demonstrated by one-way ANOVA (F = 178.6, p = 0.0001). Optical absorbance was measured using UV-visible spectrophotometry at 550 nm. Since absorbance is dimensionless, the values are reported as AU. As shown in Table 1, Group E (Negative Control) exhibited the highest mean optical absorbance (0.435 ± 0.014 AU), indicating the greatest dye extraction and microleakage, whereas Group D (Positive Control) showed the lowest mean optical absorbance (0.042 ± 0.020 AU). Among the experimental materials, Biodentine demonstrated the highest microleakage (0.192 ± 0.013 AU), followed by GuttaFlow Bioseal (0.160 ± 0.017 AU) and Bio-C Repair (0.120 ± 0.008 AU). As shown in Table 2 and Figure 1, Tukey HSD post hoc analysis revealed statistically significant differences in optical absorbance between all pairwise group comparisons (p < 0.001). As shown in Figure 2, the greatest mean difference was observed between Group D (Positive Control) and Group E (Negative Control) (mean difference = 0.3936 AU), whereas the smallest significant difference was observed between Group A (Biodentine) and Group C (GuttaFlow Bioseal) (mean difference = 0.0317 AU). These findings indicate distinct differences in microleakage among all study groups.

**Table 1:** Statistical analysis of microleakage scores and comparison among groups using one-way ANOVA f value is 178.6 (ANOVA test) p value of 0.0001 is considered significant ANOVA: analysis of variance; SD: standard deviation

Groups	n	Mean ± SD	Minimum	Maximum	95% confidence interval	
					Lower bound	Upper bound
Group A (Biodentine)	10	0.192 ± 0.013	0.175	0.212	0.183	0.202
Group B (Bio-C Repair)	10	0.120 ± 0.008	0.108	0.133	0.114	0.126
Group C (Guttaflow Bioseal)	10	0.160 ± 0.017	0.139	0.193	0.148	0.173
Group D (Positive Control)	10	0.042 ± 0.020	0.018	0.077	0.027	0.056
Group E (Negative Control)	10	0.435 ± 0.014	0.413	0.459	0.425	0.445

**Table 2:** Post hoc Tukey HSD analysis - mean microleakage score differences (intergroup comparisons) ^*^Significant p value SE: standard error; HSD: honestly significant difference

Groups	Mean difference	SE	p value	95% confidence interval	
				Lower bound	Upper bound
Group-A (Biodentine)					
vs. Group-B (Bio-C Repair)	0.07200	0.007	<0.001^*^	0.0527	0.0913
vs. Group-C (Guttaflow Bioseal)	0.03170	0.007	<0.001^*^	0.0124	0.051
vs. Group-D (positive control)	0.15050	0.007	<0.001^*^	0.1312	0.1698
vs. Group-E (negative control)	-0.24310	0.007	<0.001^*^	-0.2624	-0.2238
Group-B (Bio-C Repair)					
vs. Group-C (Guttaflow Bioseal)	-0.04030	0.007	<0.001^*^	-0.0596	-0.021
vs. Group-D (positive control)	0.07850	0.007	<0.001^*^	0.0592	0.0978
vs. Group-E (negative control)	-0.31510	0.007	<0.001^*^	-0.3344	-0.2958
Group-C (Guttaflow Bioseal)					
vs. Group-D (positive control)	0.11880	0.007	<0.001^*^	0.0995	0.1381
vs. Group-E (negative control)	-0.27480	0.007	<0.001^*^	-0.294	-0.2556
Group-D (positive control)					
vs. Group-E (negative control)	-0.39360	0.007	<0.001^*^	-0.4129	-0.3743

**Figure 2:**
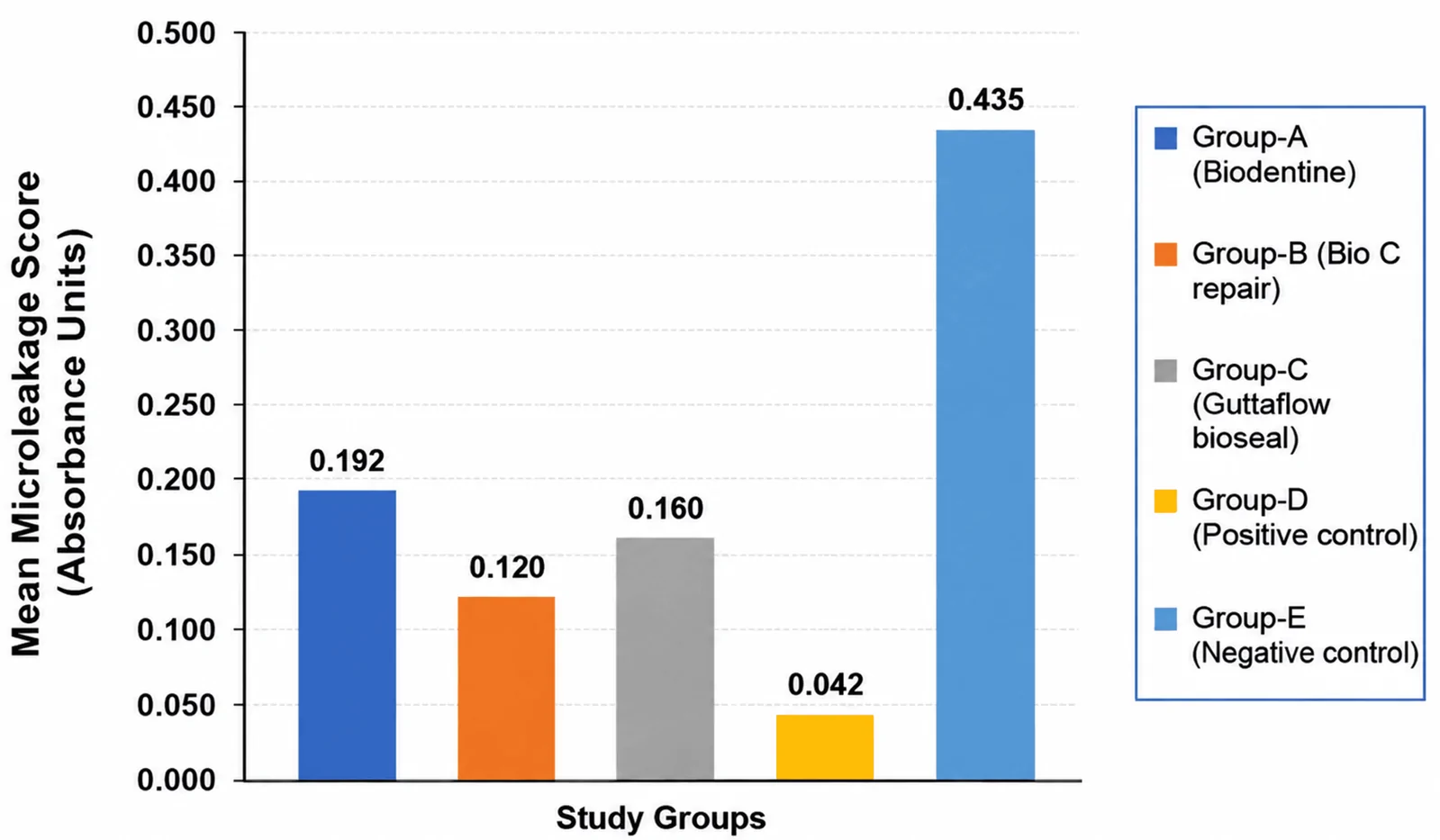
Comparison of five study groups

## Discussion

Three materials were tested in this study: Biodentine™, Bio-C Repair, and GuttaFlow Bioseal. Throughout the investigation, Biodentine™ exhibited the highest amount of microleakage among the three experimental materials. Several factors may explain the higher leakage observed with Biodentine™ in sealing strip perforations. As a calcium silicate cement, Biodentine™ may have lower flowability and adaptability than Bio-C Repair and GuttaFlow Bioseal, making it less effective in irregular perforation sites. While Biodentine™ relies on hydration and intrinsic bonding, Bio-C Repair and GuttaFlow Bioseal may provide better marginal adaptation due to their bioactive and silicone-based compositions, respectively. Additionally, slight dimensional changes and higher porosity may compromise the sealing ability of Biodentine™ compared with the other tested materials. These factors may collectively contribute to its relatively higher microleakage.

This finding is consistent with the observations of Nowicka et al., who reported that the porosity and characteristics of Biodentine™ may change when stored under dry conditions, causing gaps at the root-dentin interface and resulting in leakage. Dimensional instability and increased porosity, often associated with variations in the water-to-cement ratio, may adversely affect its sealing properties. In contrast, Bio-C Repair and GuttaFlow Bioseal demonstrated superior sealing performance, which may be attributed to their comparatively lower porosity [12].

Porosity is an important factor affecting material properties such as adsorption, permeability, and sealing ability. The increased porosity of Biodentine™ may therefore negatively influence its sealing performance compared with other root repair materials. However, Brizuela et al. concluded that Biodentine™ demonstrated superior sealing ability compared with MTA, supported by several studies highlighting its effectiveness in various dental applications [13].

Bio-C Repair is another material that has recently been investigated, with promising outcomes reported during a one-year follow-up. Studies have demonstrated favorable cell viability, cellular adhesion, and migration, while its ready-to-use formulation simplifies clinical procedures [11]. The addition of carbon nanotubes has also been reported to enhance the compressive and flexural strength of Bio-C Repair while reducing its porosity, thereby potentially improving its durability and compensating for certain material limitations [14].

In the present study, Bio-C Repair demonstrated the best performance in minimizing microleakage, with a mean absorbance value of 0.120. Its superior sealing ability compared with Biodentine™ and GuttaFlow Bioseal may be attributed to its bioactive composition, which promotes hydroxyapatite formation and enhances adaptation to dentin. Its favorable flowability may facilitate sealing of irregular perforation sites, while penetration into dentinal tubules may further reduce microleakage [15]. Minimal setting shrinkage may reduce the risk of microcrack formation, and its hydrophilic nature may help maintain sealing performance under moist conditions. In addition, its bioactivity may promote mineralization at the material-dentin interface. Although GuttaFlow Bioseal provides good adaptability because of its silicone-based composition, Bio-C Repair demonstrated superior overall sealing performance in the present study [16].

Cassiano Ferraz et al. confirmed that calcium silicate-based cements, such as Bio-C Repair, release ionic components that contribute to the formation of hydroxyapatite-like mineral deposits. This biomineralization may promote strong adhesion to dentin and improve the sealing properties of the material [16].

The biomineralization ability and minimal volumetric loss of Bio-C Repair are associated with its hydration characteristics, water absorption, and optimized particle size. It also demonstrates favorable marginal adaptation due to mineral precipitate formation at the cement-dentin interface [17]. However, acidic conditions may adversely affect its structural integrity, resulting in increased dissolution and dimensional loss, although immersion in phosphate-buffered saline may partially compensate for this through volume gain [18].

The third material tested was GuttaFlow Bioseal. Although limited evidence is available regarding its specific use in strip perforation repair, its favorable sealing characteristics make it a potentially useful material for this purpose [19,20]. Its flowable consistency and syringe-based delivery system facilitate convenient application and automatic mixing, which may improve consistency and reduce procedural errors. Incorporating bioglass particles in GuttaFlow Bioseal contributes to its bioactive properties while maintaining low solubility [21].

In the present study, GuttaFlow Bioseal demonstrated effective sealing, with a mean absorbance value of 0.160. Although its sealing performance was comparatively lower than that of Bio-C Repair, it still outperformed Biodentine™. The silicone-based composition of GuttaFlow Bioseal provides excellent flowability and adaptability, allowing it to conform effectively to irregular perforation surfaces. However, its sealing mechanism may differ from that of Bio-C Repair, which has greater biomineralization potential and may form a more favorable interface with dentin. While the high flowability of GuttaFlow Bioseal facilitates filling of intricate perforation sites, the superior stability and marginal adaptation demonstrated by Bio-C Repair in the present study may explain its lower microleakage values.

Supporting the findings of the present study, previous investigations have demonstrated minimal leakage and effective apical sealing with GuttaFlow Bioseal. These properties have been attributed to its adaptation to dentin, penetration into dentinal irregularities, and limited dimensional changes associated with moisture absorption [22]. Another study reported that GuttaFlow Bioseal expanded by 0.14% in distilled water after seven days and subsequently contracted by 0.68% after 30 days, in contrast to AH Plus [23]. Camargo et al. observed greater expansion, approximately 2.1%, in GuttaFlow Bioseal after 30 days. These findings further support its potential as a sealing material [24].

Several studies have reported that GuttaFlow Bioseal exhibits favorable structural integrity, slight dimensional expansion, and enhanced sealing properties associated with the presence of bioglass particles [25-27]. Its bioactivity and apatite-forming ability have been attributed to its calcium silicate and gutta-percha-siloxane-based composition. However, as summarized by Hegde and Arora, the silicone component may influence the wetting behavior of GuttaFlow Bioseal on root dentin, potentially limiting its spreadability. Its relatively hydrophobic nature may also influence contact angles and overall interaction with dentin surfaces [28].

An important consideration when interpreting the present findings is the difference in intended application and structural characteristics of the materials evaluated. GuttaFlow Bioseal is primarily a flowable obturation sealer, whereas Bio-C Repair and Biodentine are designed as bioceramic repair materials capable of filling and stabilizing relatively substantial defects. The flowability of GuttaFlow Bioseal may allow intimate adaptation to microscopic irregularities and narrow areas of the experimentally created strip perforation, potentially explaining its favorable microleakage performance in the present study. However, flowability and sealing ability do not necessarily indicate adequate structural performance for perforation repair. A dedicated repair cement must also maintain its position within the defect and resist displacement and mechanical stresses over time. Biodentine and Bio-C Repair are formulated for repair applications and provide a comparatively substantial material mass within the defect, together with bioactive and biocompatible characteristics. Consequently, the lower absorbance values observed with GuttaFlow Bioseal in the present study should be interpreted as an indication of favorable sealing behavior under the specific in vitro conditions rather than evidence of overall clinical superiority. Properties such as push-out bond strength, resistance to dislodgement, dimensional stability, mechanical integrity, biocompatibility, and long-term interaction with periodontal tissues remain important determinants of the clinical suitability of a perforation repair material and were not evaluated in the present investigation.

All experimental and control groups demonstrated statistically significant differences from each other, as presented in the results. This indicates that the observed differences in microleakage were associated with the specific materials and experimental conditions rather than occurring by chance. The findings were consistent with the objectives of the study and demonstrated significant differences in the sealing performance of the tested materials. Consequently, the null hypothesis, which stated that there would be no statistically significant difference among the tested materials, was rejected. Furthermore, the injectable root repair materials, Bio-C Repair and GuttaFlow Bioseal, demonstrated better sealing performance than the powder-and-liquid formulation of Biodentine™.

Limitations of the study

The present study had certain limitations. The experimental design could not completely replicate actual clinical conditions, where multiple biological and mechanical factors may influence the success of root repair materials. Although PVS was used to simulate the periodontal ligament, it could only partially reproduce the properties of the natural periodontal ligament and may have influenced stress distribution and sealing behavior. Furthermore, the long-term stability, degradation, and performance of the tested materials were not evaluated, limiting conclusions regarding their prolonged clinical behavior. Future studies should incorporate conditions that more closely simulate the clinical environment and evaluate long-term outcomes to improve the clinical relevance and reliability of the findings.

## Conclusions

Within the limitations of the present in-vitro study, Bio-C Repair and GuttaFlow Bioseal demonstrated significantly lower microleakage than Biodentine when evaluated using the dye-extraction method. These findings indicate superior sealing performance under the experimental conditions of the present study and should not be interpreted as overall clinical superiority. Clinical success of perforation repair depends on multiple factors, including push-out bond strength, biocompatibility, dimensional stability, resistance to displacement and ability to withstand functional occlusal forces. Further in-vivo and long-term clinical studies are required to establish the clinical relevance of these findings.
